# Keratoscopy

**Published:** 1879-03

**Authors:** Julia W. Carpenter


### Miscellaneous.

### KERATOSCOPY.

BY JULIA W. CARPENTER, M. D.,

*Read before the Cincinnati Academy of Medicine February 17th, 1879.*

Keratoscopy is the name given to a new method of determining the refractive power of the eye, by the play of certain lights and shadows that are made to appear upon the cornea.

No reference to an investigation by this means is found in any of the works on ophthalmology. Its discoverer is a Frenchman, M. Cuignet. He first presented the subject, and showed its importance, at the third session of the association for the advancement of science at Lille, in 1874.

Since then several articles on the subject have been contributed to Galezowski's journal, *Recueil d Ophthalmologie.* The last one, in Jan. 1878, was afterwards published in a separate pamphlet for wider circulation.

It is by Mengin, a faithful pupil of the discoverer, and one of the assistants of the immense clinic of Galezowski in Paris.

It is not claimed for this method that it obtains any new results, but simply that it has several great advantages over all other ways, and that it is one more means of making a diag nosis that is certain, rapid and very convenient, especially for children. The manner of making the examination is as follows:

The patient and light are arranged just as in an ordinary examination with an ophthalmoscope, and the simple mirror of any ophthalmoscope is used. The observer places himself, not directly opposite the eye, but a little to one side, so that the rays of light fall obliquely upon the cornea. The distance of the mirror from the eye observed is from half to three-fourths of a yard. The disk of diffused light cast upon it does not have its center correspond to the center of the cornea, but it is so directed, that one-half of it only lightens the cornea, the other half lighting the region adjoining the side of the eye examined. Two expressions are used in connection with this subject, that must not be misunderstood. When it is said such and such phenomena are seen upon the cornea, it must be remembered that that is only apparent, that it is really upon the retina that they appear. Also, by the word cornea is meant just that portion of it that corresponds in size to the opening of the pupil, as it is only through that part of its surface that the background of the eye can be seen. This use of the word is only for abbreviation. When the light is sent obliquely on the eye, in the manner described, the cornea is not uniformly transparent, but presents lights and shadows. Each type of eye has an arrangement of these lights and shadows peculiar to itself, and not only that, but in the abnormal eye these signs are more or less pronounced according to the degree of departure from the normal type.

The following is a short description of the appearances in Emmetropia; Myopia; Hypermetropia; Astigmatism and its varieties; Conical cornea; Decentered cornea.

First, the emmetropic, or normal eye. If, for example, the outer side of the right eye be illuminated in the manner described, there will appear on that side of the cornea a shadow which has the form of a triangle with rounded corners. Its base looks towards the outside, but does not reach to the outer border. Its apex points to the center of the cornea and enroaches a little upon it. All the rest of the cornea is light. If the inner, upper and lower portions, or any intermediary meridians are examined in the same way, precisely the same relative arrangement of light and shade is found.

If a movement of circumduction is given to the mirror, this dark triangle turns regularly around the center of the cornea. This picture is constant, distinct and easily recognized with a little practice.

In the report of 260 soldiers, emmetropes, examined, it is stated that in all but about ten these appearances were seen distinctly and immediately. In the ten they appeared more slowly. The shadow was not so dark, but the light had about the same degree of intensity. In the opinion of M. Cuignet such cases are a very slight deviation from the normal type, but too slight to be detected by any other method.

In myopia, or nearsightedness, there is also a typical arrangement of light and shade, as marked as in the case just described.

The light portion is at the periphery of the illuminated side, and the dark part on the side opposite, and the relative size and intensity of the light and shade depend upon the degree of nearsightedness. Three grades may be described, though of course there are many intermediate ones.

In a moderate degree the light part assumes the form of a triangle, the base reaching to the edge of the cornea and the apex almost to its center. The rest of the cornea is dark. In a very slight degree, this *light* portion is so small that it assumes the form of a little crescent, just at the edge of the cornea, almost the entire cornea being covered with a shadow, but not of a very deep tint. In a great degree of myopia the light, on the contrary, increases in size, extending from the edge of the illuminated side considerably beyond the center, leaving simply a small very dark crescent on the other side. If a movement of rotation is given to the mirror, this light portion, of whatever size, revolves around the edge of the cornea.

In hypermetropia the picture is again different, and also again as distinctly marked. The *dark* portion is at the *periphery* of the illuminated side and the light on the other, just the opposite of myopia. Here also the relative size and intensity of light and shade vary with the degree of hypermetropia, the smaller and darker the shadow, the greater the degree. Three grades here are also described.

In a feeble degree the shadow has the form of a triangle with its base directed outwards, and its apex at the center. For a moderate form the shadow is much smaller, does not reach the center, and begins to take the form of a crescent. For a great degree the shadow is still smaller, having the form of a very narrow and dark crescent at the edge of the cornea on the illuminated side, and in myopia it is the reverse.

In astigmatism keratoscopy not only diagnoses its existence, but its kind and degree. Here, however, the inspection must be more minute, as the different meridians must be examined separately. The very slight amount of astigmatism found in the normal eye is not great enough to be detected by this method.

As only the three states of refraction are possible, viz., where the rays of light come to a focus either upon the retina, before or behind it, and as in astigmatism there are at least two of these kinds, or different degrees of the same kind, it follows that the keratoscopic pictures in all the varieties must be simply combinations of those already described.

Take for example one of the commonest forms, simple myopic astigmatism, having the horizontal meridian normal and the vertical myopic.

The horizontal meridian will furnish the picture already described for the normal eye, and the vertical, that for the nearsighted eye. Now when rotation is given to the mirror, there will be a sudden change in the picture as it passes from one meridian to another. This occurring four times in making the circuit. It is easy to foresee what the picture would be in any variety, as it would always be according to the kind of ametropia to which the meridians belong.

There is no end to the variations that can occur in mixed astigmatism, but knowing the appearance for each form of ametropia, the play of light and shade can be analyzed, the direction of the meridian noticed and the diagnosis made out.

As to irregular astigmatism where the refractive power differs not only in different meridians, but in different segments of the same one, it can be recognized, but it is very difficult, and almost impossible to analyse it, so strange and irregular is the play of light and shade. Its analysis is equally troublesome, however, by the other methods, and as the difficulty can not be remedied by glasses, its detection is the thing of chief importance.

In conical cornea, the convexity being very great, the appearances are the same as in myopia, except that they are more pronounced. The light occupies almost the entire space, a thin, crescent shaped shadow only existing at the periphery, opposite the side illuminated. Decentered cornea, where the center of the convexity does not coincide with the center of the cornea, being of two kinds, the keratoscopic pictures are of two kinds also. That which is the result of some inflammatory process is usually accompanied by inequalities and opacities which prevent any constant signs being given.

In congenial decentration, where the cornea has never been the seat of inflammation it will simply present the appearances which belong to one of the three states of refraction, emmetropia, myopia, or hypermetropia, as the eye must belong to one of these classes. M. Cuignet has reported two cases of the latter kind, in both of which the decentered cornea was emmetropic.

The discoverer of this method, after great experience with it, feels authorized in affirming that the corneal peculiarities are dependent on the anomalies of refraction. He has been accustomed for a long time to make the diagnosis in this way, and he cites many interesting and peculiar cases where it was made first by this means and then confirmed by the other methods.

The explanation of the keratoscopic phenomenon, as given by Lacdolt depends upon the following principle.

When rays of light are sent into the eye from a mirror, at a distance of about half a yard, a reversed image is formed upon the retina, whether it is the normal eye or not, and the movements it makes are the opposite of those made by the object itself. In the hypermetropic eye one beholds this image just as it is, viz., upside down. But looking into the myopic eye from a certain distance upon this reversed image, it is reversed again by the eye of the observer, and an erect image is seen. This explains why in myopia and hypermetropia the corneal appearances are just the opposite of each other.

The advantages of keratoscopy are several. It is free from some of the ordinary inconveniences.

The examination being made in a dark room, the pupil is already expanded. In the usual inspection with the ophthalmoscope, the light sent into the eye is so great as to cause instant contraction of the pupil from reflex action of the irritated retina, but in this case there is almost no reflex contraction, as the light cast upon the eye is so moderate. It is rarely necessary, therefore, to subject the patient to the inconveniences of atropine.

This method is also extremely useful where the eye, from any of the various causes is over-sensitive to light. It is especially valuable in some forms of astigmatism, where it is a rapid substitute for the usual researches often long and tiresome to both patient and physician.

As before stated, it is not claimed for this method that any new results are obtained, but that it is extremely convenient and very rapid. One unacquainted with it would be surprised at a clinic to see how quickly and easily the diognosis is made by one who is skillful in its use.

It is certainly gratifying too, when a child, stupid, simply because it has never been well, can have its difficulties solved, independently of intelligent answers which it can not give. And when after several weeks wearing of suitable glasses, the child is brought back, and one is told that it is like another person with new eyes, one is repaid for the time spent in learning still another method of diagnosis.—*Lancet and Clinic.*

